# Experiences in Conducting Participatory Communication Research for HIV Prevention Globally: Translating Critical Dialog into Action through Action Media

**DOI:** 10.3389/fpubh.2016.00128

**Published:** 2016-06-22

**Authors:** Warren Martin Parker, Antje Becker-Benton

**Affiliations:** ^1^Independent Consultant, Cape Town, South Africa; ^2^FHI-360, Washington, DC, USA

**Keywords:** action research, participation, social and behavior change communication, communication for social change, mobilization, methodology

## Abstract

**Problem:**

Developing communication to support health and well-being of vulnerable communities requires a multifaceted understanding of local perspectives of contextual challenges and potentials for change. While participatory research enhances understanding, robust methodologies are necessary to translate emerging concepts into viable communication approaches. Communicators and change agents need to clarify pathways for change, barriers and enablers for change, as well as the role, orientation, and content of communication to support change. While various approaches to participatory action research with vulnerable communities have been developed, there is a dearth of methodologies that address the formulation of communication concepts that can be applied at scale.

**Methods:**

The Action Media methodology has been refined over a period of two decades, being applied to addressing HIV, related aspects such as gender-based violence, as well as broader issues, such as maternal and child health, sanitation, and malaria in Africa, The Caribbean, and Asia. The approach employs a sequence of interactive sessions involving communicator researchers and participants from one or more communities that face social or health challenges. Sessions focus on understanding audiences through their engagement with these challenges and leading to shaping of relevant communication concepts that can be linked to mobilization for change.

**Results:**

The Action Media methodology contributes to processes of shared learning linked to addressing social and health challenges. This includes determining priorities, identifying barriers and facilitators for change, understanding processes of mobilizing knowledge in relation to context, determining appropriate communication approaches, and integrating indigenous language and cultural perspectives into communication concepts. Emerging communication strategies include support to systematic action and long-term mobilization.

**Recommendations:**

Communication to address public health concerns is typically developed through expert-led didactic approaches that, at best, engage audiences at the end of the development cycle through pretesting of communication concepts. Action Media provides an alternative approach that can be utilized to inform communication by integrating community perspectives at the outset. Notwithstanding the focused engagement with small group representative of health-vulnerable subpopulations, Action Media findings have informed large-scale communication interventions. The approach is directly linked to enabling ownership, critical thinking, and mobilization of knowledge for change.

## Introduction

The development of health communication includes tensions between approaches that focus on technical efficiencies of delivering messages conceived by health experts directed to influence individual behavior change and approaches that locate health communication within socio-ecological frameworks. These latter approaches recognize that individual health is situated within a context of intrapersonal relationships, cultural practices, economic, and social conditions (1). Similarly, conceptions of health literacy – which relates to capacities to interpret health information – include approaches that emphasize individual capacity to interpret health information in comparison to approaches that consider barriers and facilitators for health, the relation between information and meaning, and communication to support critical thinking and empowerment (2). Such orientations represent variations within the extent to which audiences are drawn into the communication production cycle, with implications for the relative power between communicators and audiences (3).

While it might be expected that such divergences would evolve toward establishing clear consensus in the health communication field, or stronger guidance to new practitioners, this is not the case. Persuasive approaches that follow compliance-oriented biomedical imperatives coexist with approaches that prioritize the social and material contexts of audiences within a socio-ecological milieu. Conceptual frameworks for the latter include social and behavior change communication (SBCC) approaches, which consider the links between interpersonal factors and contextual dynamics, and communication for social change (CFSC) approaches, which draw on participatory dialog and empowerment models that trace back to the work of Paolo Freire in the 1960s (4, 5).

Persuasive approaches typically utilize mass media communication to achieve wide reach, in combination with expert-informed dialog delivered by health professionals, health promoters, or peer educators. By comparison, although SBCC and CFSC approaches may also employ mass media and expert-led dialogs among other modalities, there is a particular concern with how audiences negotiate health concepts in relation to material conditions, as well as having an interest in influencing response beyond the individual level (6). This includes placing emphasis on developing analytic skills, exploring intrapersonal relationships and challenges, and acknowledging that people are both individual and social actors in relation to health (7).

Among communication theorists, there has been concern about the emphasis placed on rational health models, which tend to be based on biomedical models, in contrast to Afrocentric frameworks that acknowledge that the meaning of health is shaped by diverse cultural factors (8). For example, in relation to HIV communication, an alternate framework was proposed through a series of critical reviews in the Journal of Health Communication (9) and later work (10), which foreground family and community, expressions of gender power, traditional beliefs, and value systems that need to be taken into account in communication production and dissemination. Concerns have also been raised about the tensions between Western emphases on individual empowerment, which coincide with a focus on media campaigns, and Afrocentric emphases on collective empowerment with a focus on dialog, group communication, and social networks (5).

While these theoretical considerations have much to do with how health communication might be effective – and approaches across the continuum are all able to demonstrate change – there is also the need to consider the development and production cycles that bring about health communication content in the first instance.

While health communication approaches oriented toward biomedical logics are premised on obtaining understanding of scientific concepts to support health, there is a tendency toward repetitive and simplistic top-down communication that is delivered at the expense of understanding the specific communication needs and contextual nuances of audiences. For example, an analysis of the perspectives of community leaders on the Ebola emergency in Liberia found that there was little need to continuously disseminate information describing Ebola etiology and transmission in a context where more complex support and guidance was needed. Gaps identified included the need to extend emphasis beyond a focus on Ebola symptoms and the risks of body washing toward addressing diverse concerns including community-level hygiene and sanitation; community involvement in surveillance; community support to quarantine and burial; support to orphans and survivors; and memorialization of victims. Communication was also noted to underutilize local languages and preferred communication modalities (11). A further specific concern was the lack of engagement with community leaders to explore such issues. Similar concerns have been raised about the state of health communication in general, where little emphasis has been placed on audience reception research (12).

In contrast, participatory approaches, which tend to be used too little in risk communication, focus on audience perspectives in the first instance, seeking to emphasize community dialog, mutual learning, solidarity and collective agency toward social action (13). Examples include the integration of community narratives and representations through methodologies such as *PhotoVoice* and participatory film production such as *Scenarios from Africa* (14). Through such participatory research processes, community members document their context through visual and audio–visual technologies, utilizing images and vignettes and contributing stories to engage with health and social issues within their immediate contexts (15). While such approaches focus on codevelopment of communication with representatives of prospective audiences, it has been observed that they may fall short in relation to sustained participation, and additionally, that resource-intensive activities with small communities are incompatible with going to scale (16).

## Methods

Against the background of theories and approaches, as well as fluidity in the field, Health Communicators are bound to apply their individual competencies and systems of communication production to health challenges. There is, however, a dearth of information on systematic methodologies for developing communication and their application and outcomes in real-world contexts.

As a contribution toward addressing this gap, we outline our experiences in the development, implementation, and refinement of a communication-focused participatory action research methodology – Action Media – that has been applied to HIV and other aspects of health communication in diverse settings and with a range of audiences over the past two decades.

Action Media is derived from principles of action research – a process involving “collaborative ways of conducting social research that systematically satisfies rigorous scientific requirements and promotes democratic and social change” (17). The methodology involves engaging with audience representatives to understand health vulnerabilities and risks, determining health and development priorities, exploring language and esthetic interests, identifying media and communication preferences, and codeveloping communication concepts that are meaningful, relevant, and appropriate to the live experiences of participants. Findings are then applied to communication strategy, refining communication products and approaches, and communicating at scale.

Action Media was developed in South Africa in the 1990s during the period when the response to an emerging HIV epidemic was constrained by the then *apartheid* state. National level response included racial stereotyping and paternalistic approaches that did not adequately consider health contexts and vulnerabilities (18, 19). At the time, there were also a number of global examples of HIV communication that were grounded in grassroots response – for instance, political and esthetic approaches that supported the HIV prevention and care among gay men in the United States during the 1980s by organizations such as ACT-UP, and community-derived concepts that were prominent in Uganda’s response to HIV. ACT-UP’s campaign included mobilizing slogans such as “silence = death” that evoked and opposed persecution and marginalization of gay men (20), while concepts such as “zero grazing” addressed the links between HIV and promiscuity in Uganda (21).

The research enquiry that led to the development of Action Media took place in the context of a South African academic study, whereby HIV-vulnerable youth living in communities in Johannesburg and Soweto were engaged to determine the extent youth could be involved in crafting HIV communication concepts (22). A particular emphasis was to explore how the power dynamics between a communication researcher and research participants could be negotiated, taking into account divergences in language, literacy, and social context. Furthermore, it was intended that trust and common purpose be established to address HIV, framed by the desire to “pursue both truth and solutions to concrete problems, simultaneously” through following an action research approach (23).

A step-wise process was followed with a group of 15 youth who were recruited on a voluntary basis, based on their interest in HIV. An extended program of participatory sessions was negotiated, and a series of five 4-h interactive sessions were facilitated by a single researcher. Emphasis was placed on informal dialog in group discussions, where participants shared their perspectives. Participants were free to communicate in their language of preference. Translation into English was provided by participants who could speak both languages. Three teams of five participants each were established to explore particular questions related to the broader enquiry and to report their views back to the large group at various stages – for example, their understanding of biomedical aspects of HIV and factors underlying HIV risk among youth. In these small groups, discussions took place without any presence of the researcher-facilitator. Maintaining the composition of the small groups throughout the sessions allowed participants to build trust and deepen the extent to which they shared skills and knowledge between themselves and with the larger group. To sustain focused participation, energizers and games were used. Role-plays were employed to interrogate the relation between ideas for change and problem-solving.

During the process, participants critically reviewed existing HIV communication materials, discussed the HIV reception environment, and related what they knew about HIV as it touched their own lives. This information was linked to capacities to negotiate HIV risks and vulnerabilities and led to sessions that involved developing poster concepts, which could be used in their community contexts. These were refined with the assistance of a graphic artist, reviewed by participants, finalized, and reproduced in small quantities.

The emerging poster concepts were closely related to participants’ environments. For example, among the concepts developed was an image depicting a condom in colors of the national flag – which had recently been adopted as part of the political transition in 1994 – with the slogan “Viva condoms.” From the perspectives of participants, this concept was linked to protecting one’s country and showing that the new democracy was committed to the health of all citizens. This poster was used by participants to support condom promotion and distribution in conjunction with rallying response at events such as World AIDS Day in their communities.

Although concerns were raised in various quarters beyond the originating community – including, for example, by government representatives who felt that the image denigrated the national flag – the poster was later subsumed into the national HIV campaign run by the Department of Health, and millions of copies were disseminated. This initial experience suggested the potential for communication concepts derived from small representative groups through Action Media, to be utilized at scale.

In the period through to the present, Action Media has been applied with diverse audiences globally, including people who inject drugs, men who have sex with men (MSM), sex workers, persons living with HIV, persons of low literacy, persons affected by gender-based violence, and various youth and adult groups. Engagements have taken place in countries in sub-Saharan Africa, The Caribbean, and China to address health-related vulnerabilities such as HIV, Malaria, reproductive health, water sanitation, and hygiene.

The methodology has largely followed its original conception, with some modifications. Facilitation by a single researcher has been expanded to include cofacilitators, translators, graphic artists, and other persons with technical skills in communication. A standardized sequence of activities that can be completed over 4 days has been adopted. All activities are carefully planned before-hand, including being outlined in a research protocol. The protocol includes a review of literature on the focal health issue in the country and local context, addresses ethical concerns, attends to recruitment and logistics, and provides an outline of activities and topics to be covered.

Implementation involves spending a day exploring health contexts and challenges, a second day exploring communication reception and developing various communication concepts, a third day that excludes participants and allows graphic artists and communication specialists to develop prototype concepts from preceding sessions, and a fourth day to review prototypes and potentials for ongoing activities.

Activities intersperse large and small group discussions, summaries and presentations of findings, energizers, and role-plays. Cultural concerns are addressed – for example, it is common in many African settings to include prayer and singing as part of meetings, and this has included integration of both Christian and Muslim faiths.

All discussions are digitally recorded and transcribed, including being subjected to further translation if necessary. Photographs are taken as part of the documentation process, with video recordings also being useful for documenting role-plays, or folk media, such as songs.

**Figure 1:**
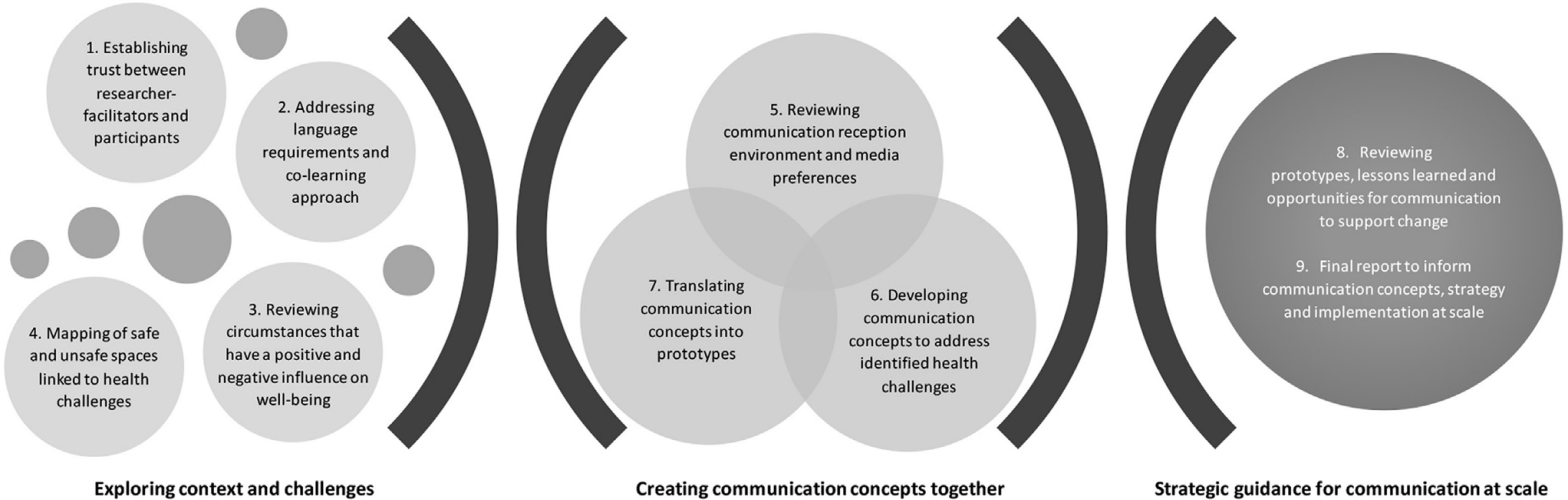
**Action Media communication development process**.

In summary, processes include (1) introductory exercises to acquaint participants and facilitator-researchers with each other, and to foster trust; (2) sharing proposed formats of interaction and co-learning, languages of preference and translation, and addressing ethical concerns including freedom of expression, mutual respect, and confidentiality; (3) review of the context and circumstances of participants lives, including aspects that have a positive and negative influence on well-being; (4) a community-mapping exercise exploring safe and unsafe spaces related to health; (5) review of the communication reception environment and media preferences, including folk media; (6) engaging with slogans, proverbs, phrases, songs, and other communication concepts relevant to addressing health challenges; (7) translation of communication concepts into prototypes; (8) Reviewing prototypes, lessons learned and opportunities for communication to support change; (9) Compiling a final report to inform communication concepts, strategy and implementation at scale (see Figure 1).

Specific resources required for implementing Action Media are modest. These include a hall with moveable seating and space for large and small group work, flip chart sheets for researcher facilitators and participants to document and share concepts, three to four digital recorders to capture large and small group proceedings, one or more cameras for audio–visual documentation, and resources necessary to produce communication prototypes. Participants are provided with refreshments and meals, and stipends are paid to cover transportation costs and recognition for time taken.

## Results

While the outcomes of Action Media have generated diverse learnings, we share, here, selected findings from work in the field drawn from published and unpublished research reports documenting Action Media processes. This research has largely been initiated by international and national non-governmental organizations to explore predetermined health concerns with specified subpopulations, contributing to the design of communication concepts, campaigns, and programs, in study countries and beyond. Throughout these implementations, Action Media has revealed links between focal health concerns and other vulnerabilities that were not apparent through mainstream research.

### Altruistic Values

Action Media was conducted to address the risks of HIV transmission through needle-sharing and unclean injecting equipment *via* sessions conducted with recent heroin users in Kunming – a Chinese city close to Asia’s opium producing “golden triangle” (24). It was found that while participants were largely aware of health risks linked to the transmission of HIV, there was an immediate concern about the need for detailed information on resuscitating friends who were at risk of dying as a result of overdosing on heroin. Sessions, then, explored ideas to support this concern leading to the development of concepts toward an illustrated leaflet that also addressed first aid in conjunction with injecting equipment safety to prevent HIV.

Follow-on sessions focused on developing slogans and images attached to heroin use, with emerging concepts being closely linked to altruistic values. These included concepts that discouraged new drug users – for example, a poster concept depicting a set of scales showing a single syringe in balance with multiple syringes with the slogan “*One injection is too much, 10,000 are never enough*” and a poster concept encouraging rehabilitation of drug users that depicted a person being pulled from the claws of a dragon (a reference to heroin) by a group of people, with the proverb “*people who help people, also help themselves*.”

### Marginalized Populations

Action Media was conducted in Jamaica and The Bahamas to address HIV-related vulnerabilities among sex workers and MSM (25, 26). Sex work and homosexuality are illegal in these countries, and both subpopulations experience social ostracism, harassment, and persecution.

Introductory Action Media sessions established that the risks of HIV infection were framed not only by limited access to HIV information and services, but also by social concerns, economic factors, and experiences of violence. For sex workers, it was necessary to be secretive about their work to ensure that their children would not be stigmatized and that their families would not reject them. Their work included vulnerability to theft of cash and personal items, coerced involvement in drug trafficking, violence and threats of violence by clients, and being manipulated, harassed, and detained by police and border authorities. Links to HIV included conducting sex work in high-risk environments, having to exchange sex to avoid detention, being forced to have sex under threat of violence, being forced to have sex without a condom, and limited access to health and social services. For MSM, risks included being rejected by their families, lack of safe spaces where they could socialize, limited access to religious institutions, sensationalist representations of homosexuality in the media, risks of physical abuse, and difficulty accessing services.

Communication concepts identified through Action Media addressed these concerns in various ways. For example, to allow for covert communication, electronic versions of slogans and images were developed for display on cell phones as an alternative to print products that could be found by authorities. Both groups identified a lack of unity and mutual support as undermining their collective interests. Social fragmentation among MSM was linked to variations in sexual preferences that defined gender and social identity, as well as lack of co-operation between men who were open about their sexual orientation and those who were secretive or “*down-low*.” This led to slogans and images promoting unity among MSM as a whole. For example, in The Bahamas, communication concepts were conceived that addressed unity between MSM such as “*Let’s not fight – Unite*” and “*Unity for the community*”. The latter slogan was incorporated into a poster and sticker concept portraying silhouettes of a group of men in rainbow colors to convey links to the gay pride movement. Communication concepts were also directed toward the external community in ways that emphasized acceptance and humanity of MSM – for example, the slogans “*Heal the world. Accept me the way I am*” and “*Who am I? Human*” attached to an image of Leonardo da Vinci’s Vitruvian man.

Sex workers identified various strategies for ensuring that they were safer that could be disseminated by word of mouth. These included sharing information about risky situations, keeping friends informed of one’s whereabouts, memorizing details of clients who picked up other sex workers, and making friends with security guards. Concepts of safety were reinforced through slogans such as “*Run away and live to see another day*” (to avoid violence) and “*Tell me how that meter running*” (to support sharing of safety concerns between sex workers).

In Jamaica, communication concepts incorporated patois and slang terminology commonly used by sex workers and MSM. Sex workers emphasized pride in their attractiveness and commitment to HIV prevention with the slogan “*Mi smart, mi sexy, mi always have mi condom*,” while for MSM, commitment to condom use was expressed in the slang phrase “*No raw baw baw*,” meaning no sex without a condom. A range of similar slogans were developed to support HIV prevention dialog in both subpopulations, and stickers and posters were identified as means for conveying and supporting such concepts.

### Contextual Communication

Action Media was conducted to address HIV vulnerabilities among fisher folk around Lake Victoria in Uganda including fishermen, commuter boat operators, fish mongers, and traders. A community-mapping exercise not only identified exposure to marijuana, alcohol, and sex work as risky, but also clarified that a key factor underpinning these risks was a sense of fatalism directly related to the possibility of drowning in the lake. This risk was associated with bad weather and storms, rudimentary equipment, and lack of life jackets. Approaches that could be developed to improve personal safety on the lake were then explored, as a precursor to confronting HIV risks when ashore. Suggested strategies included improving access to low-cost life jackets and organizing safety improvements through the involvement of the boat owner’s associations.

Regarding HIV risks, an exercise exploring slogans and poster development illustrated how instead local proverbs could be employed to provide insight and foster reflection of HIV risks. For example, the need to take steps to reduce risks was expressed in a proverb in Luganda meaning “*when you see a pothole ahead, you must swerve*,” which was linked to two visual concepts – one depicting a man with a sickly friend, and another depicting a man appearing to consider sex with a sex worker. Another concept inverted conventional constructions of masculinity that place value on men as courageous, employing humor and irony through the heading in Luganda meaning “*Be alert*,” combined with the slogan “*Cowards live longer*,” along with an image of a man with his arm around a sex worker.

Various other proverbs and slogans were identified by the participants, and a ranking exercise helped to inform potentials for integration into broader communication programming, including – for example – painting slogans on the exterior of boats and utilizing a logo that combined a life jacket and red ribbon to illustrate the links between safety on the lake and concerns about HIV. Participants also developed songs focused on preventing HIV and supporting people living with HIV that could be sung in lakeside communities, thereby allowing for community-owned deployment of concepts within locally relevant linguistic and cultural formats. In the communication initiatives that followed, a range of media were employed to support motivation and mobilization through promoting “champions,” sharing interpersonal communication scripts during “moonlight” gatherings at lakeside villages as well as in commercial areas, bars, lodges, and night clubs^1^.

### Participatory Communication Resources

While HIV communication addresses diverse audiences through various media, the specific communication needs of low-literacy audiences have not been well explored (27). To address this gap, Action Media was conducted with participants of low literacy in a rural area in South Africa.

It was found that knowledge about HIV and health was derived from a mix of sources, including media such as radio and television, as well as written materials (28). It was further established that preferences for printed items included the need to keep textual information brief, incorporate graphic or photographic elements to aid interpretation, use indigenous languages, include local idioms and proverbs, integrate interactive components, such as games and role–plays, and consider formats that did not need external facilitation.

The findings led to the development of various prototypes that were assessed in four southern African countries – the final iteration of which was a “Community Conversation Toolkit” (CCT). This included a set of playing cards incorporating HIV-related questions, throw cubes with proverbs and questions on each face, role-play cards and finger puppets to animate relationship scenarios and to present mini-drama’s, and button badges depicting proverbs and questions. The CCT tools were adapted into local language versions for eight countries by local non-governmental organizations, supported with facilitator’s and mobilizer’s guides, and packaged into a customized container, which was disseminated for implementation.

An evaluation of the CCT conducted in Malawi and Zambia explored use of the toolkit in a series of facilitated sessions with at-risk groups including community leaders, persons living with HIV, couples, faith groups, traditional sexual cleansers, and bicycle taxi drivers (29). The toolkit was found to evoke curiosity as a product of its novelty, overcoming a prevailing lack of engagement HIV communication attributed to “AIDS fatigue” by community-based health organizations and offering opportunities to engage with topics without prescriptive health message delivery. It was noted that the toolkit improved capacity to reach audiences who were not typically engaged by HIV initiatives, such as chiefs and community leaders. The use of games and role-plays, in combination with proverbs and questions, fostered a relaxed environment for informal learning – with participants indicating, for example, that use of the throw cube allowed one to “get deeply involved” as a product of reflecting on proverbs and questions in a group setting, as well as being engaged in a collective process of “finding answers.”

### Community Mobilization

To address violence against women in South Africa, Action Media was conducted in two settings to explore communication concepts for prevention as well as possible actions that could be taken by individuals and groups to address such violence (30). This latter focus had emerged as a product of a large-scale survey that established that the vast majority of respondents of both sexes were aware of gender rights and did not support attitudinal statements that endorsed violence against women (31).

The Action Media sessions highlighted the tensions between prosocial values, lack of clarity about steps that could be taken to address violence when it occurred (including within one’s family or community environment), and disincentives for taking action such as not wanting to become involved in private matters of others or fear of verbal or physical retaliation. Emerging creative concepts led to the development of a multicolor logo depicting intertwined arms and hands, with the slogan – “*Prevention in Action*.” The Action Media findings also contributed to reframing the program goal away from a focus on shifting attitudes validating violence against women – that were incorrectly assumed to be driving violence against women – toward transforming prevailing patterns of inaction to situations where action was taken whenever violence against women was encountered.

To support this initiative, partnerships were established with a range of community-based entities including women’s organizations, counseling services, and the police. These partner organizations identified personnel and volunteers who were trained to promote the concept of taking action. Communication support materials included the Action Media-derived logo and slogan on T-shirts, bags, buttons, and stickers, along with guidelines provided through booklets and leaflets. A network of “community influencers” was established who were encouraged to draw their friends and family members into processes of concerted response to violence against women. Further support to mobilization included developing an action-oriented manifesto as well as “*violence free home*” stickers, that were placed on doors and entry ways of private homes.

A system was established to document actions that had been taken, with narrative examples of these being disseminated through community storytelling events, newspaper articles, and short video clips. Emphasis was placed on allowing actions to be crafted organically rather than prescribing specific strategies for action. This approach ensured that actions were relevant to the dynamics of particular instances of violence. Over a period of 18 months, around 2,500 action narratives were documented, and it was clear that a groundswell of action had resulted in tangible changes in the two communities (32). Narratives detailed how participants had addressed gender-based violence in their personal relationships as well as addressing occurrences in their family and community environments. Many actions were also stimulated by participants who were identified through wearing branded items, to whom one could go for help. Continuous action research processes allowed participants to improve ways of organizing at community level, leading to the concept of “Violence Free Zones.” In these areas, community members conducted clean-ups and painted community spaces in the colors of the logo. Evaluation of the initiative found that the logo had come to represent concepts of togetherness and unity, including dimensions of ownership (“it belongs to us”) and action (“let’s do it”). The branded T-shirts allowed participants to identify each other – and as participants observed – contributing to a “circle of friendship” and sense of validation: “we feel like social workers of the universe.”

## Discussion

Action Media has evolved into a robust replicable methodology that has been applied in diverse contexts toward the development of health communication resources and products, while also informing communication to support subsequent social processes. The methodology includes close attention to participant empowerment, including applying specific techniques that address the power relations between researcher-facilitators and research participants. For example, researcher facilitators follow a process of open-ended enquiry that allows participants to identify and clarify their immediate concerns, while also identifying links to serious health concerns such as HIV. While questions are posed by researcher-facilitators, participants have the opportunity to pose and address questions of their own. Participant empowerment is also aided by an emphasis on informal enquiry, including opportunities for warmth and laughter derived through reflective dialogs, as well as utilization of energizers, games, and role-plays.

A particular contribution of the approach is establishing processes that allow for the research to be conducted cross-culturally and in diverse linguistic contexts – for example – through language translation during large group discussions, with discussion in small groups being entirely conducted in the formal or informal language of choice of participants without the presence of a researcher-facilitator.

Within the Action Media sequence, participants obtain a critical awareness of the circumstances of their own health through collaborative engagement and shared learning, leading to the formulation of solutions linked to communication. Exploration of underlying challenges and barriers linked to focal health issues such as HIV allows for multipronged solutions. For example, strategies to address fatalism among fisher folk related to fears of drowning were combined with an exploration of strategies to address HIV – with both supporting future health and well-being.

While it might be anticipated that engaging a small, relatively homogenous subset of participants’ representative of a particular audience would not contribute sufficient information relevant toward generalizable communication concepts, this has not been the case. Action Media experiences in diverse contexts demonstrate that findings can inform communication processes that reach other communities, as well as being relevant at national and multinational levels.

Action Media also informs communication principles. For example, while Action Media has been used to identify specific proverbs that hold meaning for participants in particular settings, it is simultaneously established that proverbs are a preferred way of making meaning in relation to health – and this is a generalizable outcome. Similarly, low-literacy audiences identified games as a preferred means of engaging with health information, leading to a range of communication development processes that shaped specific products, drawing on the identified underlying affinity for a game-like format of communication.

### Exemplars

The finding that people who inject drugs were oriented toward altruistic communication to address drug overdose among friends and peers, as well as supporting cessation of drug taking and drug rehabilitation, highlights the value of Action Media in fostering critical thinking toward problem-solving that includes addressing prosocial concerns. This contrasts with individual-focused persuasive approaches directed towards injecting drug users that address HIV only within a biomedical framework. For example, the World Health Organization’s guidance for HIV prevention, treatment, and care for people who inject drugs describes a “comprehensive package” that includes needle and syringe programs; opioid substitution therapy; HIV testing and antiretroviral treatment; condom programing; sexually transmitted infection, tuberculosis, and hepatitis prevention, diagnosis, and treatment; and targeted information education and communication (33). This singular biomedical focus contrasts with communication to mobilize and empower people who inject drugs to inform, reshape, and transform their HIV-related vulnerabilities.

Action Media with marginalized MSM and sex workers in The Caribbean exposed power relations that impeded capacities to address HIV vulnerabilities and risks. In particular, it was necessary to articulate specific strategies that could support unity within these subpopulations, as well as improving strategies for mutual support linked to personal safety. Unity was reinforced through integration of the globally recognized gay pride “rainbow” colors, as well as incorporating references to concepts of “ideal man,” which is expressed in the use of da Vinci’s ideally proportioned Vitruvian man. Similarly, the Jamaican sex workers’ slogan – “Mi smart, mi sexy” – additionally contributes to a sense of unifying pride and validation through attachment to concepts of being clever and sensible, while also being sexually desirable.

The use of slang terminology and indigenous phrases – including sexualized terminology – by the Jamaican participants stands apart from conventional communication that employs non-indigenous, authoritative and formal language to address health. For both these vulnerable subpopulations, communication concepts were developed that could be shared covertly and openly to support community mobilization, with agency to communicate in relation to health situated with MSM and sex workers themselves, rather than as recipients upon whom health communication is enacted.

Fisher folk in Uganda have been identified by health communicators as being “hard-to-reach” as a product of their mobility (34), while health communication directed toward this subpopulation has tended to involve unproductive stereotypes, such as portraying fisher folk as being reckless risk-takers (35). Action Media illustrated how values that were conventionally attached to masculinity of fisher folk, were critiqued through concepts validating cowardice, over courage by using the slogan “cowards live longer.” Related concepts also underpinned a sense of solidarity and mutual support among fisher folk that portrayed friends looking out for each other – in contrast to health communication that appeals mainly to individual interests.

As a form of qualitative research, Action Media efficiently teases out underlying concerns and challenges through critical reflection, thereby working against assumptions that HIV-vulnerable subpopulations have an affinity for risk-taking. Such motivations may be overlooked in research approaches that assume vulnerable persons lack insight into the underlying determinants and motivations for risky behavior.

Action Media to address the health communication needs of low-literacy audiences provided insights into affinities and preferences for communication approaches that allowed for critical reflection through participatory interaction. These would have been difficult to conceive without the insights and guidance obtained through an interactive process that negotiated the relationship between communication practices and potentials for health communication. Furthermore, the evaluation of the CCT highlighted the capacity of Action Media to identify conceptual approaches to non-traditional communication that supported critical thinking in group settings (36, 37). This contrasts with the emphasis of some peer education approaches that are weighted toward information delivery and didactic methods (38).

With respect to community mobilization through the Prevention in Action program, Action Media contributed to the foundational communication elements, with subsequent cycles of action research reinforcing individual and group responses to address gender-based violence. While communication products such as T-shirts, bags, buttons, and stickers did not convey detailed information in and of themselves, they substantially supported a deeper understanding of response that was regularly enacted. For example, the sticker bearing the Prevention in Action logo and text reading “violence free home” communicates simple information in the first instance, but is transformed into a complex system of meaning when it is attached to the door of a private residence. This includes meaning for the residents themselves, as well as visitors and observers. Such meaning is also not fixed by the specific communication product, but continuously shaped by its relation to its situational value in the context of a particular residence that is different to another. Similarly, participants who wore the branded T-shirts came to represent people who were ready to assist and who obtained social validation as a product of crafting and enacting solutions to problems. The logo also supported the construction of ideological meanings as a product of the colors being painted onto private and public spaces within violence free zones.

These examples illustrate the importance of developing communication products and resources that allow for meaning to be developed and shaped over time and in relation to context. This process contrasts with health communication that seeks to achieve common comprehension and preferred fixed meaning through communication activities and products within campaign-defined timeframes (39).

## Recommendations

Participatory health communication and social change theories provide an alternative to persuasive health communication approaches that seek efficient information delivery of health knowledge grounded in biomedical constructs and rationale. Action Media, thus, aligns with theories that integrate cultural perspectives and values into health communication (10), as well as theories that emphasize participation, critical consciousness, and social mobilization (5).

While the value of communication theory is to inform fundamentals of communication practice, there is a dearth of literature describing methodologies that translate theoretical principles into practical approaches for developing communication resources, approaches, and products. The deference of health communicators to linear transfer of biomedical knowledge to support health can be understood as a product of the simplicity of their production processes. For example, preconceptions about knowledge gaps frame the orientation of such communication, with emphasis given to compliance and adherence to health behaviors and practices in contrast with allowing health circumstances, culture, and context to be negotiated in relation to health imperatives. The experiences of Action Media implementation illustrate how emerging communication products link to activities that address power imbalances, foster critical thinking, enable ownership, and mobilize knowledge for change.

Action Media provides a systematic and replicable approach to inform the development and implementation of health communication programs through critical engagement with audience representatives. It has largely been employed at the outset of health communication programs and has informed both initial communication design and subsequent implementation. A particular value is that the approach is cost and time-efficient, with findings being relevant to immediate communities as well as communication programs that are carried out at scale.

## Author Contributions

WP conceived and drafted the manuscript outline. WP and AB-B jointly drafted and revised the manuscript. Both authors read and approved the final manuscript.

## Conflict of Interest Statement

The authors declare that the research was conducted in the absence of any commercial or financial relationships that could be construed as a potential conflict of interest. The reviewer (JH) and handling editor declared a current collaboration and the handling editor states that the process nevertheless met the standards of a fair and objective review.

## Footnotes

^1^Communication for Healthy Communities (CHC), Quarterly Report to USAID, unpublished project report, 2015.
